# Supplementary material for: Identification of photoperiod-induced specific miRNAs in the adrenal glands of Sunite sheep (Ovis aries)
Source: Front Vet Sci. 2022 Jul 22;9:888207. doi: 10.3389/fvets.2022.888207 (PMC9354845; doi:10.3389/fvets.2022.888207)
Supplement: Supplementary file 1 [file Data_Sheet_1.ZIP › Supplementary file/miRDeep2 file.zip!/miRDeep2 file/Novel_Structure/LP42A2/Novel_610.pdf]

| | | |
|---|---|---|
| Provisional ID | : | NC_019484.2_38427 |
| Score total | : | 0.5 |
| Score for star read(s) | : | -1.3 |
| Score for read counts | : | 0 |
| Score for mfe | : | 0.2 |
| Score for randfold | : | 1.6 |
| Score for cons. seed | : | |
| Total read count | : | 150 |
| Mature read count | : | 150 |
| Loop read count | : | 0 |
| Star read count | : | 0 |

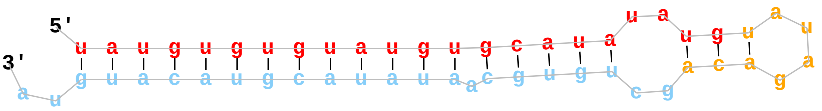


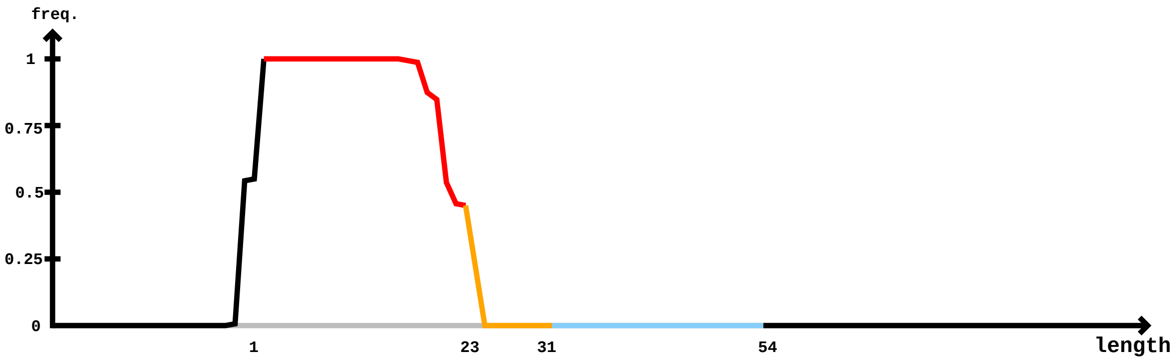


Mature Star

```
5'- uuauuaugaggauuucuauauauguguguaugugcauauauguauagacagcugugcaauauacguacauguauguauacaaaugucauuggugugcauuagguaacuucuc   -3'    exp
    .......((((...((((((((((((((((((((((((..(((....)))..))))).)))))))))))))))(((((((.(((...))).))))))).))))...))))..     reads    mm    sample
    .................auauauguguguaugugUaua..........................................................................     1        1     seq
    ..................uauauguguguaugugG.............................................................................     1        1     seq
    ..................uauauguguguaugugUa............................................................................     1        1     seq
    ..................uauauguguguaugugUau...........................................................................     17       1     seq
    ..................uauauguguguaugugUaua..........................................................................     3        1     seq
    ..................uauauguguguaugugUauau.........................................................................     47       1     seq
    ..................uauauguguguaugugUauaua........................................................................     12       1     seq
    ...................auauguguguaugugUauauau.......................................................................     1        1     seq
    ....................uauguguguaugugcauauaug......................................................................     1        0     seq
    ....................uauguguguaugugUauauaug......................................................................     64       1     seq
    ....................uauguguguaugugUauauaugu.....................................................................     3        1     seq
```
